# Experimental Research of Drying Characteristic of Red Banana in a Single Slope Direct Solar Dryer Based on Natural and Forced Convection

**DOI:** 10.17113/ftb.59.02.21.6876

**Published:** 2021-06

**Authors:** Elavarasan Elangovan, Sendhil Kumar Natarajan

**Affiliations:** Department of Mechanical Engineering, National Institute of Technology Puducherry, 609609 Karaikal,; UT of Puducherry, India

**Keywords:** single slope solar dryer, red banana drying, drying kinetics, moisture ratio correlation, moisture diffusivity, activation energy

## Abstract

**Research background:**

Traditionally, open sun drying method serves to dry the products for long time preservation. Solar drying is also employed to minimise the drying time to achieve the required moisture content. This method inherently contains complex heat and mass transfer mechanisms, which makes difficult to describe drying kinetics at the micro level.

**Experimental approach:**

In this paper, research is carried out to investigate the drying of 5 mm thick slices of red banana (*Musa acuminata* ’Red Dacca’) in a single slope solar dryer based on natural and forced convection. Based on the experiments, a new semi-empirical thin layer drying kinetics is proposed and compared with other existing models. The proposed model with the correlation coefficient (R^2^) of 0.997 is in very good agreement with other well-known models. Based on the model, we calculated the moisture diffusivity and activation energy of the red banana drying process.

**Results and conclusions:**

It was found that the moisture diffusivity of the red banana samples was in the range 0.87-1.56·10^-9^ m^2^/s for natural convection solar drying and 0.84-2.61·10^-8^ m^2^/s for forced convection solar drying. The activation energy of the red banana varied from 24.58 to 45.20 kJ/mol for passive and 22.56 to 35.49 kJ/mol for active drying. Besides, we carried out energy and exergy analyses of red banana in the dryers and found that the average exergy losses in the forced and natural convections were 16.1 and 6.63 kJ/kg and the average exergic efficiency of the natural and forced convection dryers was 57.7 and 70.9%, respectively.

**Novelty and scientific contribution:**

A single slope direct solar dryer was designed and built to maintain the desired temperature for a specified period in both natural and forced convection mode. A novel drying kinetics model with higher correlation coefficient (R^2^) than the other drying kinetic models is proposed for the preservation of red bananas.

## INTRODUCTION

Banana is one of the most popular fruits around the world. Among all the developing countries, India is one of the largest producers of banana (*1*). The banana is abundantly grown and harvested in almost all tropical countries. Almost 40% of its production is lost in the post-harvesting process due to improper handling and unconventional way of storage. There are 1000 varieties of banana available in and around the world. Among them, in India, the popular varieties are Baby, Dwarf Cavendish, Giant Cavendish, Red, Pisang raja, Manzano, Burro, Barangan, Gold finger and Saba banana. Among all these bananas, red banana has good nutritional value but is costly. Hence, we tried to study the drying kinetics of red banana. Arivazhagan and Geeta (*2*) reported that 6.5% of the bananas are wasted at the wholesale distribution points in Tamil Nadu, India. One of the studies reported that the lifetime of bananas is 2 to 9 days in a refrigerator, 2 to 3 months in a freezer and 6 to 12 months when dried. Therefore, the drying of banana is preferable as it is a reliable method to improve its life and avoid wastage. Koua *et al*. (*3*) developed a mathematical model for thin layer drying of mango, banana and cassava in a passive solar dryer. In order to validate their study, we tested the proposed model in seven empirical and semi-empirical models available in literature. Henderson and Pabis drying mathematical model was in close agreement with experimental results. Da Silva *et al*. (*4*) dried whole Brazilian bananas at temperatures from 40 to 70 °C in the convective dryer to develop the mathematical model to describe its drying phenomenon. It was concluded that Page and Silva models were in close agreement with the drying kinetics. Öngen *et al*. (*5*) studied the drying characteristics of green olives in hot air dryer at varied air temperatures from 40 to 70 °C with a constant air speed of 1 m/s. They found that the green olives attained equilibrium moisture content with good sensory acceptance in 22 h, and also mentioned that the dried olives can be stored for up to one year without any deterioration.

Akbulut and Durmuş (*6*) analysed the energy and exergy of mulberry in a forced convective dryer based on five different mass flow rates ranging from 0.014 to 0.036 kg/s. They mentioned that the energy utilization ratio and exergy loss decreased with the increase of mass flow rate. Omolola *et al*. (*7*) modelled drying characteristics of Luvhele banana during thin layer drying in an oven to calculate the effective diffusivity. They compared six mathematical drying models and concluded that two-term model was the best for the prediction of drying characteristics of Luvhele banana. Doymaz (*8*) experimentally determined the drying characteristics of banana in a hot air dryer with the air temperatures varied from 50 to 80 °C at a wind speed of 2.4 m/s. They concluded that the Page and logarithmic models were in good agreement with other existing models. The effective diffusivity of banana sample was in the range from 0.74 to 2.14·10^-10^ m^2^/s and its activation energy was 32.65 kJ/mol. Mierzwa *et al*. (*9*) compared hybrid and microwave drying of carrot samples pretreated with ultrasonically assisted osmotic dehydration. They observed that the combination of microwave and infrared waves with hot air drying allows shorter period of drying carrot samples with lower energy consumption.

Hempattarasuwan *et al*. (*10*) focused on the drying kinetics of cayenne pepper and the effects of the drying treatments in both solar and electrical tray dryers on capsaicin content in whole pods and cut samples of cayenne pepper. The authors observed that cayenne pepper dried faster in the electrical than in the solar dryer due to stable drying temperature. They also observed that Midilli-Kucuk model was more suitable for whole pods and Page model for cut samples of cayenne pepper. Akpinar (*11*) constructed the convective dryer for drying red peppers at different air temperatures from 54 to 70 °C with a constant air velocity of 1.5 m/s. It was reported that exergetic efficiency varied from 67.28 to 97.92% with increased drying time. Dandamrongrak *et al*. (*12*) examined the effect of four different pretreatments such as blanching, chilling, freezing and combined blanching and freezing of banana on the drying kinetics at 50 °C air temperature with inlet air velocity of 3.1 m/s and 10-35% relative humidity in a heat pump dryer. They found that drying rate was enhanced with the freezing pretreatments. Also, the two-term exponential drying model was the most suitable for describing this drying process, with banana moisture diffusivity 4.3-13.2·10^-10^ m^2^/s. Using the first and second law of thermodynamics, Midilli and Kucuk (*13*) carried out energy and exergy analyses of dried shelled and unshelled pistachio in a solar cabinet dryer.

Simo-Tagne *et al*. (*14*) developed the mathematical model to evaluate the convective and mass transfer coefficients of dried ebony wood in a natural convection indirect solar dryer. The obtained convective heat and mass transfer coefficients during drying of the sample varied from 0.25 to 5.5 W/(m^2^·K) and from 1.0·10^-8^ to 5.5·10^-8^ m/s, respectively. Komes *et al*. (*15*) studied the quality improvement of dried strawberries by the addition of two different sugars such as trehalose and sucrose. They found that the combination of trehalose with freeze drying resulted in the better quality than of the other sample.

Based on the literature review, very few research works analysed the energy and exergy efficiencies of the banana drying. Most researchers concentrated on the drying process of commonly available species of yellow banana and no research has been carried out on red banana. Hence, in this article, we have attempted to study the drying characteristics of red banana and to estimate the moisture diffusivity and activation energy in a single slope direct solar dryer under passive and active mode. Besides, we compared the results with open drying, as well as other existing drying models and developed a new correlation between the drying moisture ratio and drying time.

## MATERIALS AND METHODS

### Experimental set-ups

**Fig. S1:**
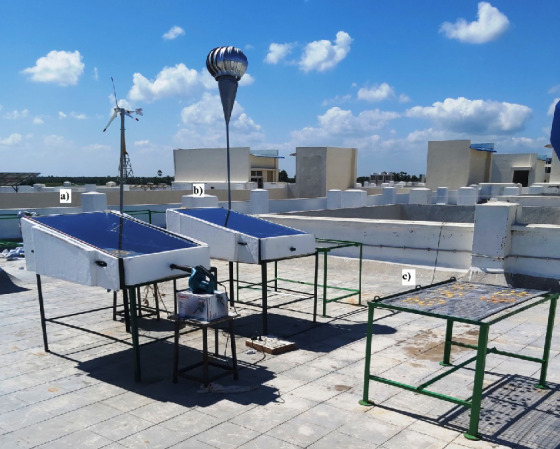
Photograph of the experimental set-up for banana drying: a) forced convection, b) natural convection single slope solar dryer and c) open sun drying

The experiments were performed in a single slope direct solar dryer, which has a trapezoid shape with base dimensions of 1290 mm×850 mm with two different heights of 500 and 260 mm, respectively. Sides of the solar dryer were constructed using a dual 1.5 mm thick galvanized sheet with a gap of 50 mm between the inner and outer walls of the sheets, which were filled with coconut husk to reduce the heat loss from the sides of the dryer. Top of the dryer was covered with a flat transparent glass of 5 mm thickness with an inclination of 10.9° (latitude of the place, Karaikal District). Outer surfaces were enclosed in an insulation chamber that consists of two layers of thermocol each 25 mm thick with an air gap of 25 mm between them to reduce significantly heat loss. An aluminium mesh of dimensions 1190 mm×750 mm was constructed and placed inside the dryer at about 50 mm vertically from the absorber plate of the dryer. This mesh served as the plate for placing red banana slices for drying. The inlet air was supplied to the dryer through a 22 mm diameter mild steel pipe placed horizontally in the lower side of the drying chamber. A similar outlet pipe was placed vertically on the opposite end of the dryer (perpendicular to the inlet of the system). The entire drying chamber was placed on a stand of mild steel L brackets of 25 mm thickness, dimensions 1290 mm×1000 mm with a height 750 mm. Two similar set-ups were made. One was used for natural convection and the other one for forced convection with a blower (model M4000B; Makita, Bangalore, India), providing air velocity of 1.5 m/s. In addition, open drying method experimental set-up was made and the same quantity of red banana was dried to compare the drying time of forced and natural convection. The photographs of natural convection, forced convection single slope dryer and open sun drying experimental set-ups are shown in Fig. S1. Eight K-type thermocouples (with the accuracy of ±0.1 °C) were attached at different locations to measure the surface, air and atmospheric temperatures continuously. A Hukseflux pyranometer (model SR20-TI; Hukseflux, Delft, The Netherlands), with the accuracy of ±10 W/m^2^, was used to measure the intensity of global radiation. The thermocouples and the pyranometer were connected to a data acquisition unit (model 34972A; Keysight, New Delhi, India) to measure the temperature and global radiation (*16*, *17*).

### Sample preparation

The red bananas (3 kg) were purchased from the local market in Karaikal, Puducherry, India. The skin was peeled off on the day of the experiment and the samples were cut into 5 mm thick cylindrical shapes with a uniform diameter of 50 mm. The samples were then carefully placed in the dryers for open sun drying, forced and natural convection drying from 9.00 am to 5.00 pm. Parameters monitored for the analysis were energy, exergy, effective moisture diffusivity and activation energy of the red banana samples.

### Exergy and energy analyses

The analyses of red banana were carried out based on the first and second law of thermodynamics, where the solar dehydration of red banana is considered as a steady flow process. The drying of red banana involves heating and humidification in the dryer chamber for effective removal of the moisture from the samples. The conservation of mass and energy in steady-state flow can be used to equate the processes (*5*). The equations that govern the conservation of mass and energy, respectively, are as follows:


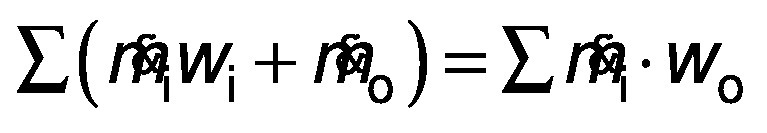



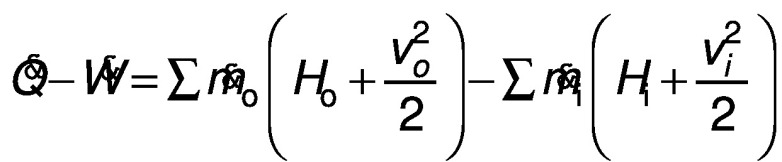


where *Q̇* is net heat rate (kW), *Ẇ* is net power (kW), *ṁ*_i_ is mass flow rate of the inlet (kg/s), *ṁ*_o_ is mass flow rate of the outlet (kg/s), *w*_o_ is humidity of the outlet air (g^-1^), *w*_i_ is humidity of the inlet air (g^-1^), *v*_i_ is velocity of the inlet air (m/s), *v*_o_ is the velocity of the outlet air (m/s), *H*_i_ is the enthalpy of the inlet air (kJ/kg) and *H*_o_ is the enthalpy of the outlet air (kJ/kg).

The relative humidity of the solar dryer chamber is an important factor to control the drying samples. The amount of relative humidity inside the solar chamber highly influences the drying rate and time. The relative humidity (*Φ*) of the system can be computed by using the following equation:


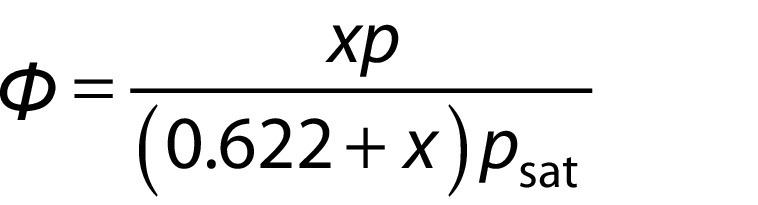


where *Φ*is relative humidity (%), *x* is specific humidity (kg/kg of dry air), *p* is the pressure in the atmosphere (kPa) and *p*_sat_ is saturation pressure of air (kPa).

The enthalpy of the dehydrating air inside the dryer is computed by using the following equation:


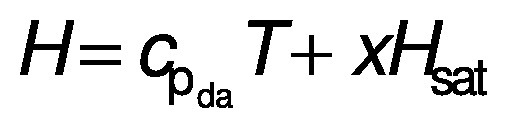


where *c*_pda_ is specific heat of drying air (kJ/(kg·K)), *T* is temperature of drying air (K) and *H*_sat_ is saturation enthalpy of air (kJ/kg).

The energy utilization ratio is a measurement of the amount of energy used in comparison to the total energy that could be used by the system. The *E*_ur_ is important to calculate the amount of energy utilized by the dryer to perform its function and to calculate the amount of energy that the system does not utilize:


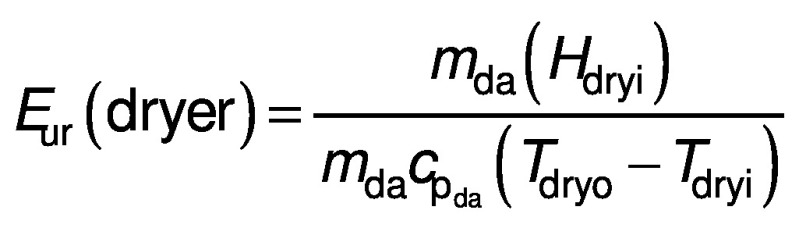


where *E*_ur_(dryer) is the energy utilization ratio of the dryer (%), *H*_dryi_ is the enthalpy of the air at the inlet (kg/s), *m*_da_ is the mass flow rate of the drying air (kg/s), *T*_dryo_ is the temperature of the outlet air (K) and *T*_dryi_ is the temperature of the inlet air (K).

In order to quantify the losses from the dryer, the exergy is also calculated. The calculation of exergy is based on the second law of thermodynamics under steady state by balancing the different forms of energy present inside the system using the first law of energy (*18*, *19*). The equation for exergy inside the dryer can be given as follows (*20*):


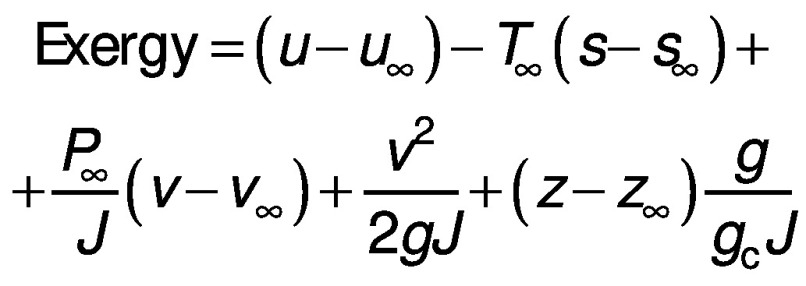


where ∞ is ambient condition, *u* is internal energy (J), *s* is specific entropy (kJ/kg K), *J* is constant, *v* is work (J), *g* is gravitational acceleration (m/s^2^), *z* is vertical datum (m) and *g*_c_ is constant.

Based on the solar dryer and source terms, the above equation is deduced to:


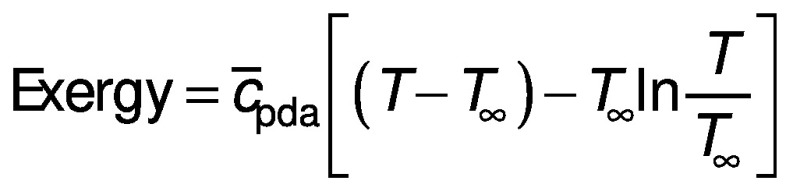


The amount of exergy in and out of system can be estimated by changing the parameters of Eq. 7 depending on the inlet or outlet condition. By applying this method, the amount of exergy inflow and outflow are computed. The exergy loss of the system can be calculated by analysing the difference between the inflow and outflow of exergy in the system:


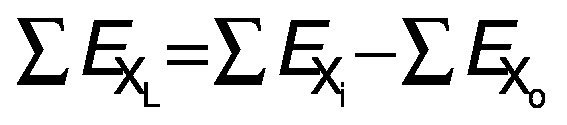


where *E*_XL_ is exergy loss (kJ/kg), *E*_Xi_ is exergy inflow (kJ/kg) and *E*_Xo_ is exergy outflow (kJ/kg).

The proposed solar dryer exergetic efficiency (*η*_EX_) is calculated by taking the ratio of the difference between the exergy inflow and exergy loss of the dryer to the exergy inflow of the dryer (*21*):


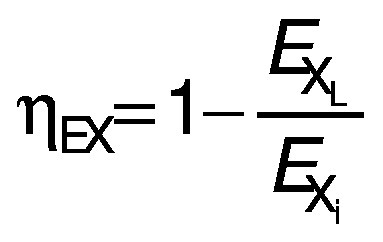


### Drying model and estimation of its parameters

The drying kinetics of thin layer of red banana is evaluated in a developed single slope dryer in active and passive mode. Then, the moisture ratio is given as:


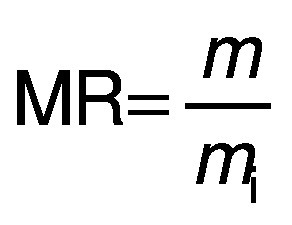


Where MR is the moisture ratio of red banana samples (%), *m* is the moisture content of red banana samples (g) at particular time, and *m*_i_ is the initial moisture content of red banana sample (g).

Based on the moisture ratio, the drying parameters (moisture diffusivity and activation energy) can be estimated. The procedure for determining the moisture diffusivity is given below.

### Effective moisture diffusivity

Moisture diffusivity is used to estimate the diffusion of moisture during dehydration. The dehydration occurs in three stages; first a uniform drying time, followed by a falling rate period of drying, and the final stage when the moisture moves from the centre to the surface (*22*). Fick’s second law of diffusion is widely used for prediction of the moisture transfer during the falling rate period of drying.


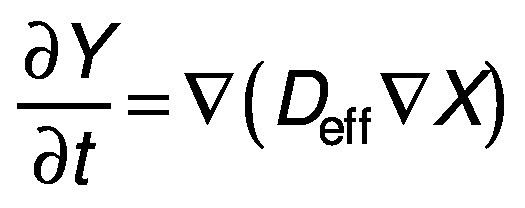


where *X* is the moisture content (g), *D*_eff_ is the moisture diffusivity (m^2^/s), ∇ is the gradient operator and *t* is time (h).

The general solution for the above equation can be obtained for different geometries (cube and cylinder respectively) and written as appropriated by the given boundary conditions (*23*):


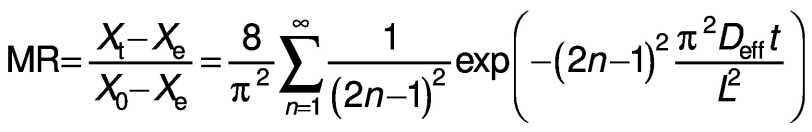



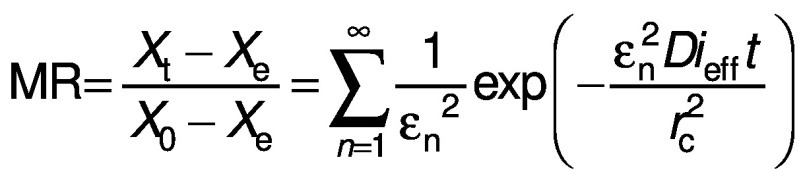


where *X*_e_ is the equilibrium moisture ratio (%), *X*_t_ is the moisture ratio at a particular period (%), *X*_0_ is the initial moisture ratio (%), *r*_c_ is the radius of the sample (mm), *L* is the thickness of the sample (mm), n is the positive integer (without units), *ε*_n_ is root of Bessel function (without units) and π is a constant.

The derived equations can be simplified by considering the first terms of the equations to compute moisture diffusivity (*24*):


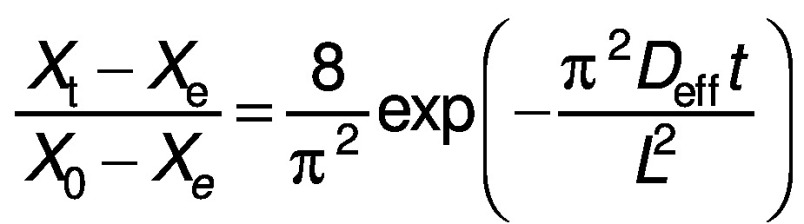



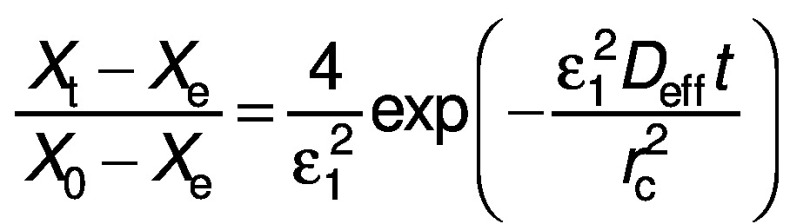


The equations can be further simplified by taking natural logarithm on both sides to make the equation logarithmic:


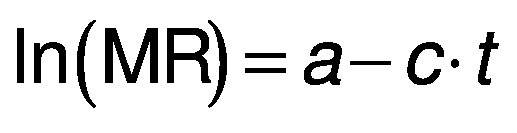


where constant *c* is 
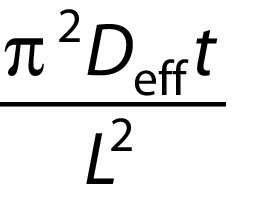
for a cube and 
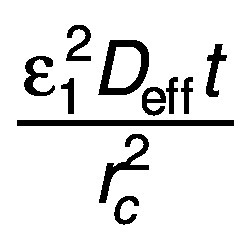
for a cylinder.

The final equation can be represented as an Arrhenius type equation:


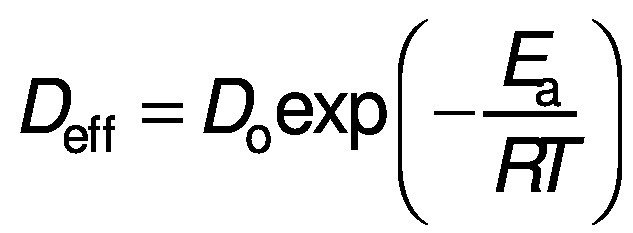


where *a* is a constant, *E*_a_ is the activation energy (kJ/mol) and *R* is gas constant (J/mol K).

### Activation energy

The activation energy of the red banana drying is the minimum of energy required for the dehydration to occur. The prediction of activation energy of red banana drying is an important task to minimise the supply of required energy. The activation energy of convective dehydration can be calculated from Eq. 17 by taking a natural log on each side of the equation:


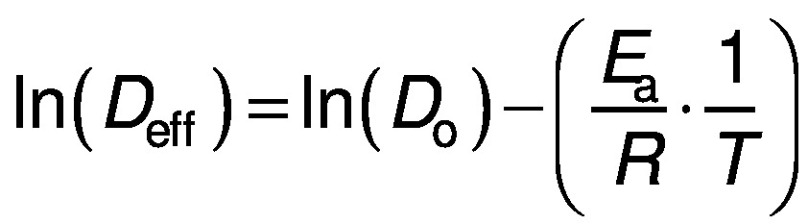


*E*_a_ of the banana can be determined by plotting a graph between ln(*D*_eff_) and 1/*T* having a slope *k* (*25*). Then, the activation energy can also be calculated as:


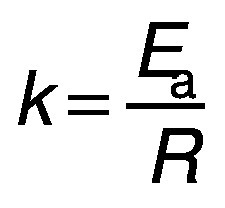


where *k* is a constant.

### Statistical and error analysis

Three statistical criteria, namely root mean square deviation (RMSD), correlation coefficient (R^2^) and reduced chi-square (χ^2^) are calculated using Data fit v. 8.0 software to validate the goodness of the fit (*26*). The best fit of the drying curve has higher correlation coefficient value and lower RMSD value. Statistical analysis was carried out to assess the consistencies between the measured and predicted values. The standard error (SE), R^2^, χ^2^ and RMSD were calculated as follows (*27*, *28*):


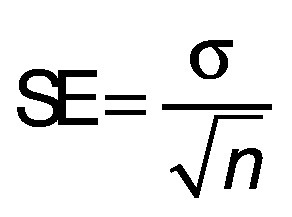



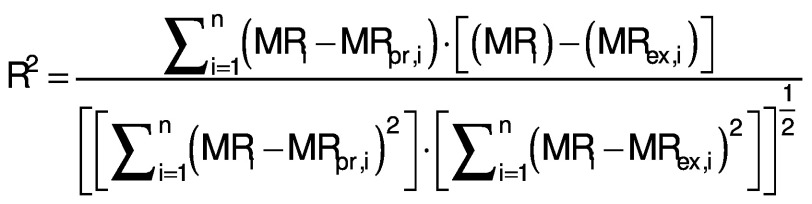



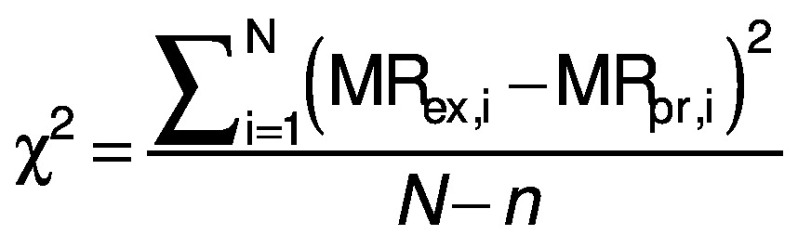



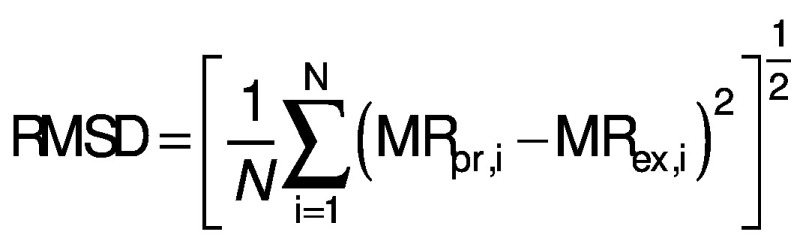


where SE is the standard error (%), *σ* is standard deviation (without units), MR_i_ is moisture ratio (%), MR_(pr,i)_ is the predicted value (%), MR_(ex,i)_ is the experimental value (%), and *N* is the number of observations.

The error analysis (w_r_) of the experimental data depends on various factors such as environmental conditions (x_1_), instrument error (x_2_) and observation (x_3_). The moisture content, solar radiation and temperature were measured using mass balance, pyranometer and thermocouple for open dehydration and solar dehydration of red banana. Table S1 gives the error values calculated by using the following equation (*29*, *30*):


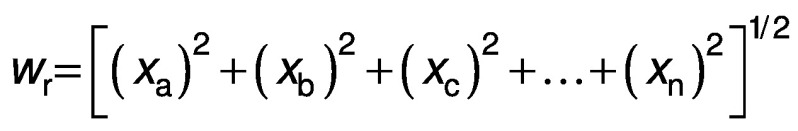


where *w*_r_ is the error analysis, *x*_a_ represents environmental conditions, *x*_b_ is instrument error and *x*_c_ is observation.

The measured uncertainty for temperature and solar radiation was ±0.05 °C and ±5.7 W/m^2^, respectively. Considering the above parameters, the uncertainty of drying rate was ±0.08 kg/s and kinetic parameters (moisture diffusivity and activation energy) of drying red banana in a single slope direct solar dryer were about ±0.42 m^2^/s and ±0.18 kJ/mol, respectively (Table S1).

## RESULTS AND DISCUSSION

**Fig. 1:**
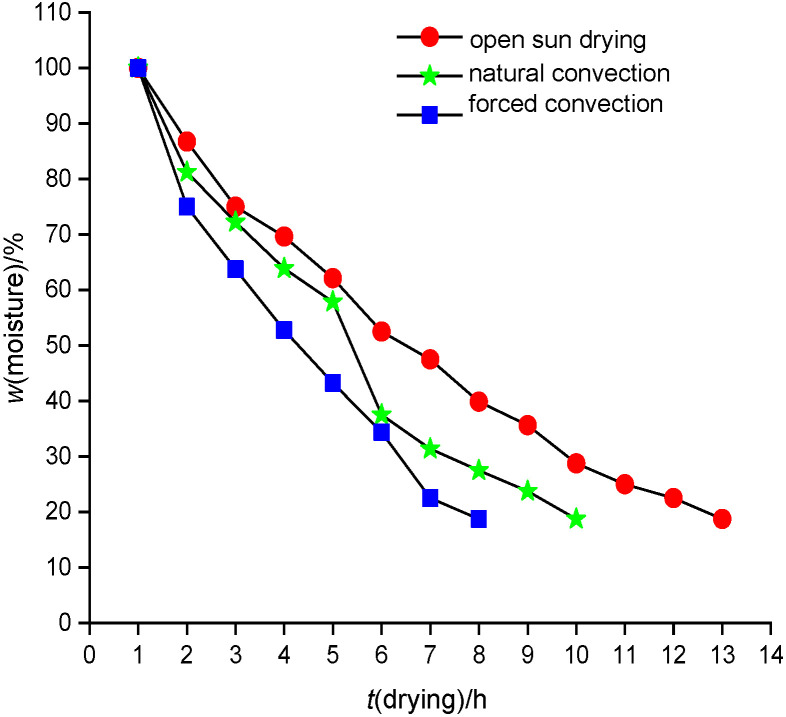
Variation of moisture ratio of the samples with the drying time during open sun, natural and forced convection drying

Extensive experiments were carried out to estimate the moisture ratio of the red banana during open drying in active or passive mode. On 10 May 2020, three experiments were performed to compare the red banana drying rates. The same quantity (one kg) of the samples was used in each experiment. The investigations were carried out from 9.00 am to 5.00 pm under clear weather. The global radiation, surface and air temperature were recorded continuously from 9.00 am to 5.00 pm. The rate of dehydration curve was obtained. Fig. 1 shows the variation of moisture ratio of the red banana with dehydration period under forced convection, natural convection and during open sun drying.

**Fig. S2:**
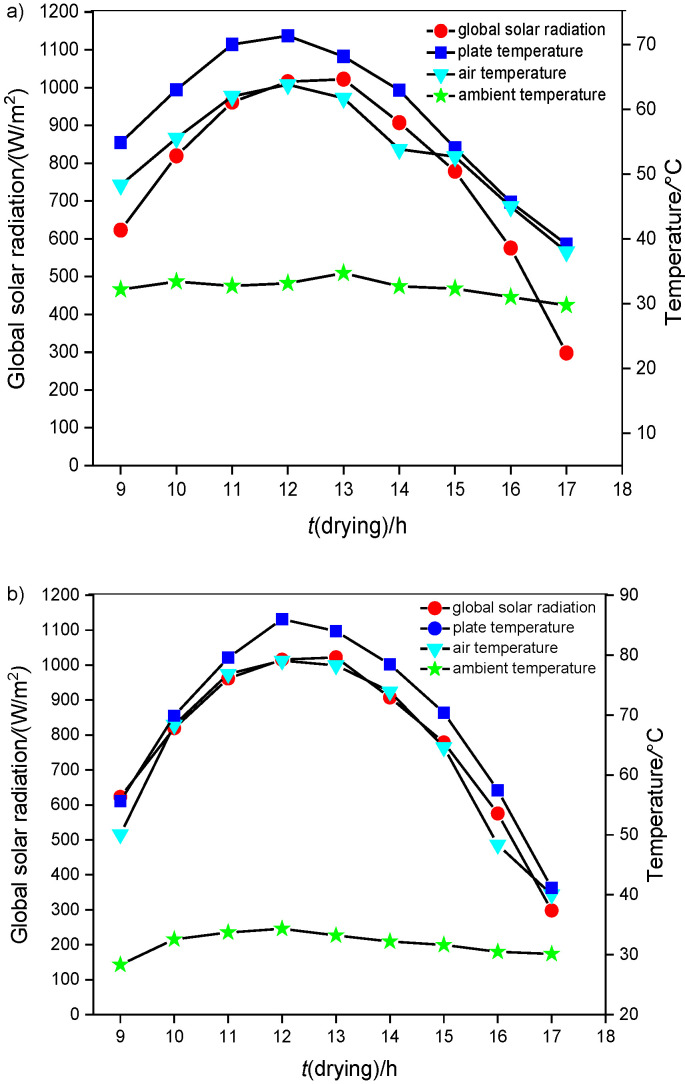
Variation of solar radiation, ambient temperature, drying air temperature, absorber plate temperature with the drying time of the samples: a) natural convection dryer and b) forced convection dryer

From Fig. 1 we were able to determine that the reduction in moisture content was higher during the active solar drying than passive solar drying and sun drying. The effectiveness of moisture removal from the samples was also enhanced with the use of forced solar dryer. It was noted that to achieve the same moisture mass fraction of 18.75%, forced convection drying was 28.5% faster than natural convection drying in a single slope direct solar dryer and 71.4% faster than open sun drying. The maximum average absorber plate and air temperatures developed in the forced convection solar dryer were 83 and 79.1 °C, respectively. Fig. 1 also shows that the rate of moisture removal was higher in the active than in the passive solar dryer and open sun drying. This is primarily due to the movement of the forced convection currents over the entire surface of the red banana samples in forced convection and the hot air penetrates through the entire surface of the samples. This movement of convection currents and hot air penetration into the samples was very slow in the natural convection and open sun drying. This obviously led to slow reduction rate of moisture content. This is evident by observing the variation of the plate, air and ambient temperature for different values of global radiations (Fig. S2).

It was observed that when maximum global radiation was 1029 W/m^2^ and ambient temperature 34.3 °C, under natural convection mode, the maximum average plate temperature was 71.3 °C and maximum average air temperature inside the dryer was 63.8 °C. Similarly, in the forced convection mode, the maximum average plate temperature and air temperature inside the dryer were 83 and 79.1 °C, respectively. It was evident that during the active drying, due to the presence of air current of 1.5 m/s, the plate and air temperature were higher than during the natural convection drying.

### Comparison with other existing moisture ratio models

Literature review shows that most of the researchers developed thin layer drying kinetics of the yellow banana and separate moisture ratio models. The list of mathematical models for determination of moisture ratio is given in Table 1 (*31*-*36*).

**Table 1:** List of mathematical models

Model name	Mathematical model	Reference
Newton	MR*=*exp(-k*t*)	(*31*)
Page	MR=exp(-k*t*^n^)	(*32*)
Modified Page	MR*=*exp[-(k*t*)^n^]	(*31*)
Henderson and Pabis	MR=a exp(-k*t*)	(*33*)
Logarithmic	MR=a exp(-k*t*)*+*c	(*34*)
Midilli-Kucuk	MR*=*a exp(-k*t*^n^)+b*t*	(*35*)
Wang and Singh	MR=*M*_0_+a*t*+b*t*^2^	(*36*)

Based on this study of active and passive drying (Fig. S1), a new moisture ratio model was developed. The proposed mathematical model was based on the Fick’s law of diffusion. The proposed semi-empirical moisture ratio correlation for red banana drying was:


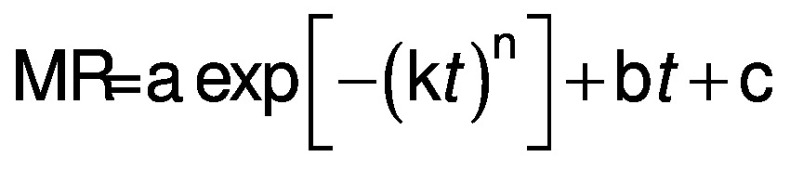


where a=3.02225 and 1.23559, b=0.07711 and -0.05846, c=-2.03566 and -0.23589, k=0.07685 and 0.02300, n=1.02043 and 0.48056 in passive (natural convection drying at 53 °C) and active (forced convection drying at 64 °C) mode, respectively, and *t* is time of drying in h.

**Fig. 2:**
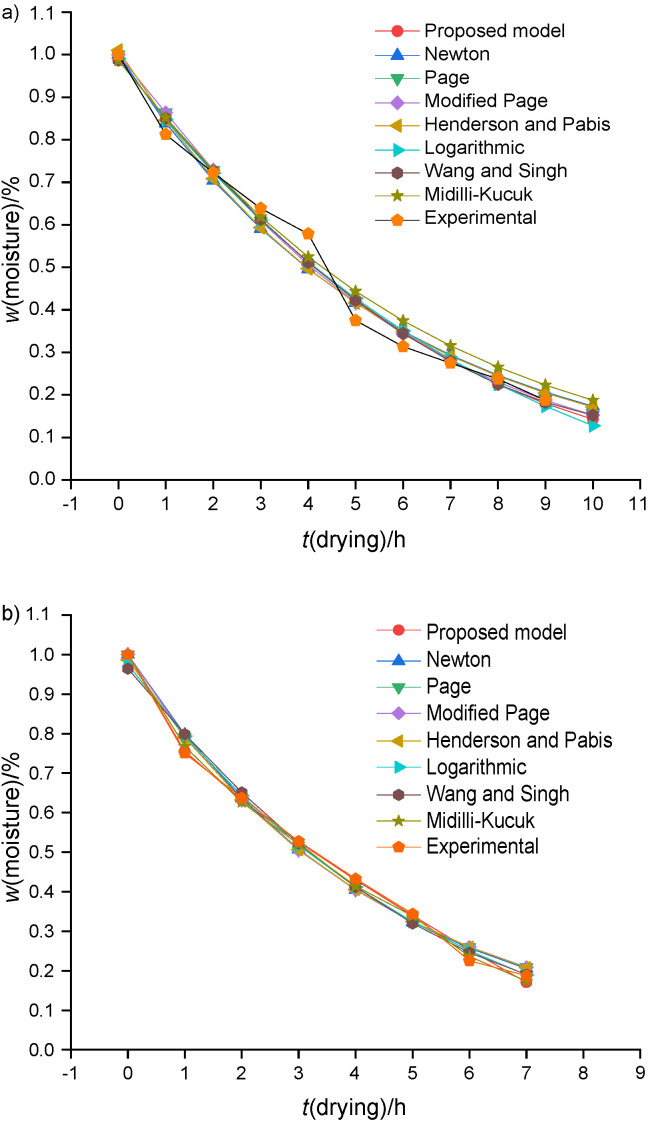
Comparison of the proposed model with other drying models from the literature (*31*-*36*) for: a) natural convection dryer, and b) forced convection dryer

Based on the existing mathematical moisture ratio models and constants, the moisture ratio in red banana was predicted for different drying time (h) for both passive and active dryer. Graphs in Fig. 2 also compare the mathematical moisture ratio model with the experimentally obtained moisture ratio for both passive and active dryers.

**Fig. S3:**
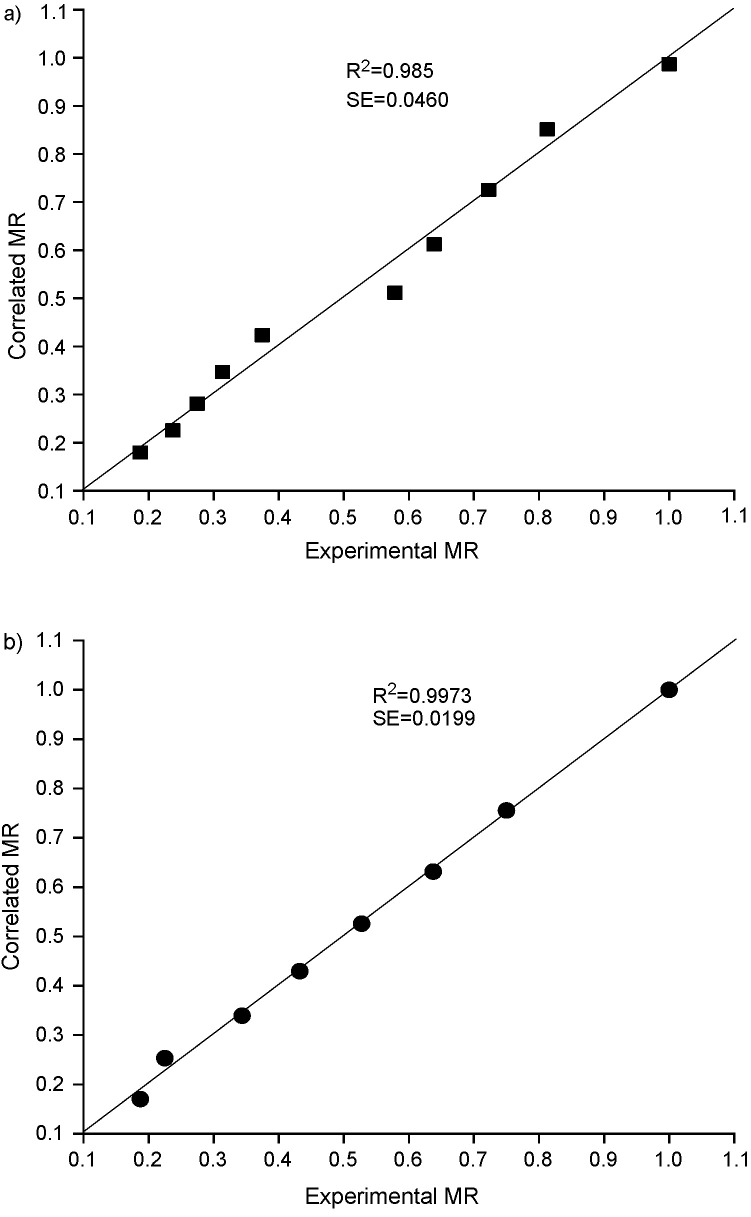
Parity plot for experimental moisture ratio (MR) *vs* correlated moisture ratio: a) natural convection dryer and b) forced convection dryer

In order to compare the proposed model with other existing mathematical models, correlation coefficient (R^2^) was calculated. The correlation coefficient (R^2^) is considered as the primary parameter to check the compatibility of the experimental data with the developed model (*37*-*39*). The values of R^2^, root mean square deviation (RMSD) and chi-square (χ^2^) for both natural and forced convection are shown in Table 2 and Table 3. We observed that the developed model is in good agreement with other existing mathematical models according to the maximum values of R^2^, χ^2^ and RMSD which are: 0.985 and 0.997, 0.003 and 0.009, and 0.0640 and 0.0873 for natural and forced convection drying respectively. Many researchers have reported similar results, where R^2^ for plantain banana (*3*), Cavendish banana (*4*), banana cv. Luvhele (*7*), papaya (*40*) and Cardaba banana (*41*) slices was found to be 0.99. Therefore, it was observed that the newly proposed semi-empirical thin layer drying kinetics model is a relatively good one for predicting drying kinetics of red banana slices. Thus, similar to other existing moisture ratio models, the present model can also be used effectively to determine the moisture ratio in red banana. In addition to that, parity plot was made for experimental moisture ratio and correlated moisture ratio for both natural and forced convection drying of red banana (Fig. S3). The correlation coefficient (R^2^) and standard deviation (S.D.) for the parity plot of natural and forced convection drying respectively were: 0.985 and 0.997 for R^2^ and 0.046 and 0.0199 for S.D.

**Table 2:** Model constants with their R^2^, reduced chi-square (χ^2^) and root mean square error (RMSE) values for natural convection drying at 53 °C

Model	Constant	R^2^	χ^2^	RMSE	Reference
Newton	k=0.17518	0.9798	0.0003	0.0640	(*31*)
Page	k=0.14681 n=1.10768	0.9830	0.0000	0.0592	(*32*)
Modified Page	k=0.17691 n=1.10768	0.9830	0.0000	0.0592	(*31*)
Henderson and Pabis	k=0.17744 a=1.01024	0.9800	0.0001	0.0658	(*33*)
Midilli-Kucuk	a=0.98631 b= -0.00687 k=0.13946 n=1.05740	0.9839	0.0000	0.0536	(*35*)
Logarithmic	k=0.12678 a=1.20046 c=-0.210228	0.9840	0.0000	0.0495	(*34*)
Wang and Singh	M_0_=0.98718 a=-0.14244 b=0.00589	0.9851	0.0000	0.0547	(*36*)
Proposed model	a=3.02225 b=0.07711 c=-2.03566 k=0.07685 n= 1.02043	**0.9846**	0.0001	0.0528	

**Table 3:** Model constants with their R^2^, reduced chi-square (χ^2^) and root mean square error (RMSE) values for forced convection drying at 64 °C

Model	Constant	R^2^	χ^2^	RMSE	Reference
Newton	k=0.22633	0.9901	0.0009	0.0868	(*31*)
Page	k=0.23324 n=0.97855	0.9902	0.0000	0.0873	(*32*)
Modified Page	k=0.22592 n=0.97855	0.9902	0.0000	0.0873	(*31*)
Henderson and Pabis	k=0.22252 a=0.98668	0.9906	0.0000	0.0840	(*33*)
Midilli-Kucuk	a=0.99971 b=-0.06038 k=0.20362 n=0.48450	0.9977	0.0000	0.0696	(*35*)
Logarithmic	k=0.18057 a=1.09485 c=-0.12004	0.9921	0.0000	0.0726	(*34*)
Wang and Singh	*M*_0_=0.96432 a=-0.17490 b=0.00919	0.9900	0.0000	0.0851	(*36*)
Proposed model	a=1.23559 b=-0.05846 c=-0.23589 k=0.02300 n=0.48056	**0.9977**	0.0000	0.0562	

**Fig. 3:**
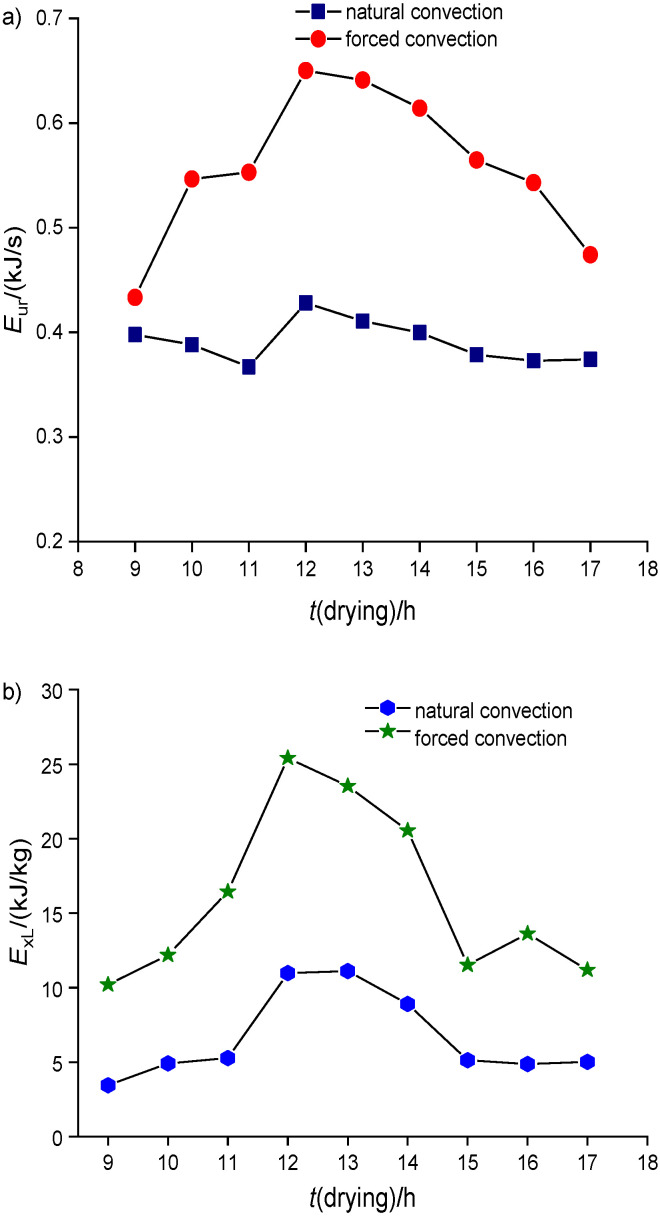
Variation in drying time of the red banana samples: a) energy utilization ratio and b) exergy loss

In addition to the above-mentioned experimental analyses of passive and active drying, *E*_ur_, exergy loss and the exergic efficiency were predicted from experimental data based on Eq. 5. The *E*_ur_ was predicted for both passive and active drying. Fig. 3a shows the variation of *E*_ur_ with dehydration period for passive and active drying. It was found that maximum *E*_ur_ was reached at the time of high insolation. The maximum obtained *E*_ur_ for natural and forced convection drying was 0.428 and 0.65 respectively. From the magnitude of the values, it was understood that the forced convection dryer efficiently sustained the *E*_ur_ due to constant airflow and effective moisture removal from the system. Apart from that, the exergy loss was also predicted to examine the wastage of energy that could have been utilized for drying. The variation of exergy loss with dehydration period in natural and forced convection dryer is observable in Fig.3b.

**Fig. 4:**
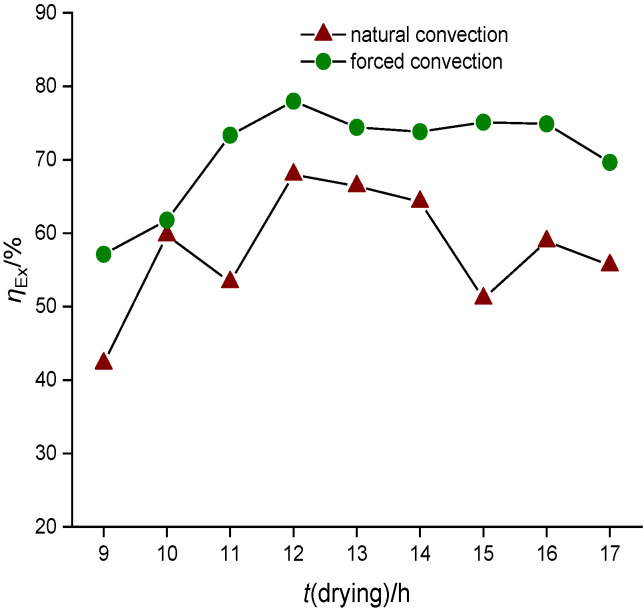
Variation of exergetic efficiency with the drying time of red banana samples

From the Fig. 3b, it was noted that exergy loss is higher in forced convection than in natural convection dryer. In the passive and active mode, the maximum exergy loss was about 11.11 and 25.42 kJ/kg respectively. This is because in natural convection dryer, the air was not admitted with uniform velocity, which allowed longer retention time of humid air before ventilation from the dryer and led to lower exergy loss than in the forced convection dryer. Also using the second law of thermodynamics, the exergetic efficiency was predicted for passive and active drying as per Eq. 9. The variation of the exergetic efficiency of passive and active drying of red banana samples is observable in Fig.4. The maximum exergetic efficiency of natural and forced convection dryer was 67.97 and 77.97%, respectively. This is due to the retainment of moisture content of the samples in the dehydrating chamber for a shorter period than during the natural convection drying. Hence, the exergetic efficiency of forced convection is higher than of natural convection dryer. Based on the exergetic efficiency, one can understand the thermodynamic characteristics of solar drying both in large and small-scale drying chamber. However, the major problem of process scale-up of the solar dryer is achieving the high temperature, which causes deterioration of the colour parameters of the sample.

The important parameters such as moisture diffusivity and activation energy were also predicted based on the experimental drying data of red banana using Eqs. 17 and 19. The moisture diffusivity depicts the rate of removal of moisture from the red banana. The least energy required for drying the sample is known as activation energy. Based on Eqs. 17 and 19, the predicted ranges of effective moisture diffusivity for the open sun drying, passive and active drying were: 3.86·10^-11^ to 1.10·10^-10^, 8.74·10^-10^ to 1.56·10^-9^ and 8.43·10^-9^ to 2.61·10^-8^ m^2^/s, respectively. In general, the value of moisture diffusivity falls in the range of 10^-9^ to 10^-11^ m^2^/s for fruits, vegetables and grains (*42*, *43*). The *D*_eff_ value obtained for mushroom samples (9.619·10^–10^ to 1.556·10^–9^ m^2^/s), pomegranate seeds (0.74·10^–10^ to 52.5·10^–10^ m^2^/s) and sweet potato (9.32·10^–11^ to 1.76·10^–10^ m^2^/s) were similar to previous research (*44*-*46*). Besides, our research group reported earlier the moisture diffusivity of pretreated ivy gourd from 7.94·10^–8^ to 3.37·10^–10^ m^2^/s (*47*).

**Fig. 5:**
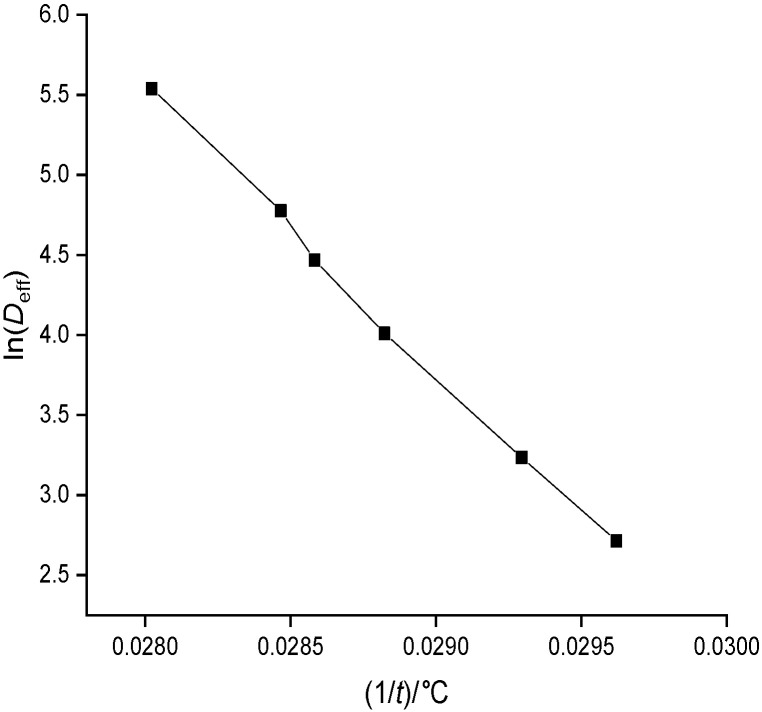
Variation of ln(*D*_eff_) with temperature

Fig. 5 shows the variation of natural logarithm of effective moisture diffusivity with temperature. Variation of moisture diffusivity is almost linear. Similarly, the range of activation energy was found to be from 29.05 to 56.53 kJ/mol for sun drying, 24.58 to 45.20 kJ/mol for passive and 22.56 to 35.49 kJ/mol for active drying. The range of moisture diffusivity and activation energy for red banana is in close agreement with other fruits reported in the literature (*48*).

## CONCLUSIONS

The experimental analysis of red banana was carried out to develop the semi-empirical correlation between thin layer drying kinetics and moisture ratio in passive and active mode of drying in solar dryers. The results of drying time were compared with open sun drying. In order to achieve the same equilibrium moisture ratio percentage of 18.75%, forced convection drying was 28.5% faster than natural convection drying and 71.4% faster than open sun drying. The proposed semi-empirical correlation between thin layer drying kinetics and moisture ratio was in very close agreement with other existing models, with correlation coefficient for natural convection drying of 0.9846 and forced convection drying of 0.9977.Based on the uncertainty of the temperature (±0.05 °C) and solar radiation (±5.7 W/m^2^), the uncertainty of drying rate and kinetic parameters for the single slope direct solar dryer was found to be ±0.08 kg/s and ±0.42 m^2^/s and ±0.18 kJ/mol, respectively. Besides, moisture diffusivity and activation energy for red banana were found and the obtained values were within the range of fruit values. In addition to the above, energy and exergy analyses served to predict the losses from the system. In order to compare the dehydration characteristics of the red banana with other fruits, we compared the experimental results with other well-known models for broader scientific analysis. We observed that the drying characteristics of red banana are in good agreement with other well-known models. Thus, the developed single slope direct solar dryer can be effectively used for drying the agricultural products and the proposed semi-empirical moisture ratio correlation for forced and natural convection can also be used to analyse the thin layer drying kinetics of food products containing fruits and vegetables. Furthermore, our intention is to extend this study to analyse the drying rate of red banana indifferent dipping solutions.

## Figures and Tables

**Table S1:** Uncertainty analysis during drying experiment of red banana

Parameter	Range	Uncertainty value
K type thermocouple	-270 to 1250 °C	±0.05
J type thermocouple	0 to 750 °C	±0.03
Air velocity	0 to 5 m/s	±0.14
Relative humidity of air	0 to 100%	±0.14
Moisture quantity	0 to 1000 g	±0.001
Global solar radiation	0 to 1650 W/m^2^	±5.77
Moisture diffusivity	-0.0 to 3.02 m^2^/s	±0.42
Activation energy	0.0 to 2.05 kJ/mol	±0.18
Drying rate	0.002 to 0.24 kg/s	±0.08

## References

[1] Horticultural statistics at a glance 2015. Ministry of Agricultue and Farmers Welfare, Govermnemt of india, New Delhi, India: Oxford University Press; 2015. Available from:http://nhb.gov.in/PDFViwer.aspx?enc=3ZOO8K5CzcdC/Yq6HcdIxC0U1kZZenFuNVXacDLxz28=.

[2] Arivazhagan R, Geetha P. Analysis of supply chain wastage for banana at wholesale distribution points in Tamilnadu. Int J Eng Technol. 2018;7(3.12):53-6. https://doi.org/10.14419/ijet.v7i3.12.15862

[3] Koua KB Fassinou WF Gbaha P Toure S. Mathematical modelling of the thin layer solar drying of banana, mango and cassava. Energy. 2009;34(10):1594–602. 10.1016/j.energy.2009.07.005

[4] da Silva WP Silva CMDPS Gama FJA Gomes JP. Mathematical models to describe thin-layer drying and to determine drying rate of whole bananas. J Saudi Soc Agric Sci. 2014;13(1):67–74. 10.1016/j.jssas.2013.01.003

[5] Öngen G Sargın S Tetik D Köse T. Hot air drying of green table olives. Food Technol Biotechnol. 2005;43(2):181–7.

[6] Akbulut A Durmuş A. Energy and exergy analyses of thin layer drying of mulberry in a forced solar dryer. Energy. 2010;35(4):1754–63. 10.1016/j.energy.2009.12.028

[7] Omolola AO Jideani AIO Kapila PF. Modeling of thin layer drying characteristics of banana cv.Luvhele. Bulg J Agric Sci. 2015;21(2):342–8.

[8] Doymaz I. Evaluation of mathematical models for prediction of thin-layer drying of banana slices. Int J Food Prop. 2010;13(3):486–97. 10.1080/10942910802650424

[9] Mierzwa D Kowalski SJ Kroehnke J. Hybrid drying of carrot preliminary processed with ultrasonically assisted osmotic dehydration. Food Technol Biotechnol. 2017;55(2):197–205. 10.17113/ftb.55.02.17.4942 28867949 PMC5569343

[10] Hempattarasuwan P Somsong P Duangmal K Jaskulski M Adamiec J Srzednicki G. Performance evaluation of parabolic greenhouse-type solar dryer used for drying of cayenne pepper. Dry Technol. 2020;38(1–2):48–54. 10.1080/07373937.2019.1609495

[11] Akpinar EK. Energy and exergy analyses of drying of red pepper slices in convective type dryer. Int Commun Heat Mass Transf. 2004;31(8):1165–76. 10.1016/j.icheatmasstransfer.2004.08.014

[12] Dandamrongrak R Young G Mason R. Evaluation of various pre-treatments for the dehydration of banana and selection of suitable drying models. J Food Eng. 2002;55(2):139–46. 10.1016/S0260-8774(02)00028-6

[13] Midilli A Kucuk H. Energy and exergy analyses of solar drying process of pistachio. Energy. 2003;28(6):539–56. 10.1016/S0360-5442(02)00158-5

[14] Simo-Tagne M Bonoma B Bennamoun L Monkam L Léonard A Zoulalian A Modeling of coupled heat and mass transfer during drying of ebony wood using indirect natural convection solar dryer. Dry Technol. 2019;37(14):1863–78. 10.1080/07373937.2018.1544144

[15] Komes D Lovrić T Kovačević Ganić K Gracin L. Study of trehalose addition on aroma retention in dehydrated strawberry puree. Food Technol Biotechnol. 2003;41(2):111–9.

[16] Natarajan SK Elavarasan E. Experimental investigation of drying potato for Karaikal climatic condition. IOP Conf Ser Earth Environ Sci. 2019;312:012021. 10.1088/1755-1315/312/1/012021

[17] Natarajan SK Sankaranarayanasamy K Ponnusamy S Kavya Chowdary V Kumar J Rahul D Experimental comparative study on reduction in the moisture content of cucumber in a double slope solar dryer with open sun drying method. J Phys Conf Ser. 2019;1276:012054. 10.1088/1742-6596/1276/1/012054

[18] Bejan A. Advanced engineering thermodynamics. Hoboken, NJ, USA: John Wiley&Sons, Inc; 2016. https://doi.org/10.1002/9781119245964

[19] Szargut J, Morris DR, Steward FR, editors. Exergy analysis of thermal, chemical and metallurgical processes. Hemphshire, NY, USA: John Wiley& Sons, Inc; 1988.

[20] Ahern JE. The exergy method of energy systems analysis. New York, NY, USA: John Wiley & Sons; 1980.

[21] Verkhivker GP Kosoy BV. On the exergy analysis of power plants. Energy Convers Manage. 2001;42(18):2053–9. 10.1016/S0196-8904(00)00170-9

[22] Dincer I Dost S. An analytical model for moisture diffusion in solid objects during drying. Dry Technol. 1995;13(1–2):425–35. 10.1080/07373939508916962

[23] Crank J. The mathematics of diffusion. London, UK: Oxford University Press; 1975.

[24] Zogzas NP Maroulis ZB. Effective moisture diffusivity estimation from drying data. A comparison between various methods of analysis. Dry Technol. 1996;14(7–8):1543–73. 10.1080/07373939608917163

[25] Barrozo MAS Souza AM Costa SM Murata VV. Simultaneous heat and mass transfer between air and soybean seeds in a concurrent moving bed. Int J Food Sci Technol. 2001;36(4):393–9. 10.1046/j.1365-2621.2001.00470.x

[26] DataFit, v. 8.0, Oakdale Engineering, Okdale, PA, USA; 2018. Available from: https://downloads.silicon.co.uk/20670/datafit-80/.

[27] Toğrul IT Pehlivan D. Mathematical modelling of solar drying of apricots in thin layers. J Food Eng. 2002;55(3):209–16. 10.1016/S0260-8774(02)00065-1

[28] Sarsavadia PN Sawhney RL Pangavhane DR Singh SP. Drying behaviour of brined onion slices. J Food Eng. 1999;40(3):219–26. 10.1016/S0260-8774(99)00058-8

[29] Moffat RJ. Describing the uncertainties in experimental results. Exp Therm Fluid Sci. 1988;1(1):3–17. 10.1016/0894-1777(88)90043-X

[30] Holman JP. Experimental methods for engineers. New York, NY, USA: McGraw Hill;1994.

[31] Akoy EOM. Experimental characterization and modeling of thin-layer drying of mango slices. Int Food Res J. 2014;21(5):1911–7.

[32] Vega A Fito P Andrés A Lemus R. Mathematical modeling of hot-air drying kinetics of red bell pepper (var.Lamuyo). J Food Eng. 2007;79(4):1460–6. 10.1016/j.jfoodeng.2006.04.028

[33] Radhika GB. VSatyanarayana SV, Rao DG. Mathematical model on thin layer drying of finger millet (*Eluesinecoracana*). Adv J Food Sci Technol. 2011;3(2):127–31.

[34] Kaur K Singh AK. Drying kinetics and quality characteristics of beetroot slices under hot air followed by microwave finish drying. Afr J Agric Res. 2014;9(12):1036–44. 10.5897/AJAR2013.7759

[35] Ayadi M Ben Mabrouk S Zouari I Bellagi A. Kinetic study of the convective drying of spearmint. J Saudi Soc Agric Sci. 2014;13(1):1–7. 10.1016/j.jssas.2013.04.004

[36] Özdemir M Devres YO. The thin layer drying characteristics of hazelnuts during roasting. J Food Eng. 1999;42(4):225–33. 10.1016/S0260-8774(99)00126-0

[37] Kassem AS. Comparative studies on thin layer drying models for wheat. In: Bartali EH, editor: Proceedings of the 13th International Congreson Agricultural Engineering; 1998 February 2-6, Rabat, Morocco: ANAFID; 1998.

[38] O’Callaghan JR Menzies DJ Bailey PH. Digital simulation of agricultural drier performance. J Agric Eng Res. 1971;16(3):223–44. 10.1016/S0021-8634(71)80016-1

[39] Verma LR Bucklin RA Endan JB Wratten FT. Effects of drying air parameters on rice drying models. Trans ASAE. 1985;28(1):296–301. 10.13031/2013.32245

[40] Yousefi A Niakousari M Moradi M. Microwave assisted hot air drying of papaya (*Carica papaya* L.) pretreated in osmotic solution. Afr J Agric Res. 2013;8(25):3229–35. 10.5897/AJAR12.180

[41] Olawoye BT Kadiri O Babalola TR. Modelling of thin-layer drying characteristic of unripe Cardaba banana (*Musa* ABB) slices. Cogent Food Agric. 2017;3(1):1290013. 10.1080/23311932.2017.1290013

[42] Madamba PS Driscoll RH Buckle KA. The thin-layer drying characteristics of garlic slices. J Food Eng. 1996;29(1):75–97. 10.1016/0260-8774(95)00062-3

[43] Babalis SJ Belessiotis VG. Influence of the drying conditions on the drying constants and moisture diffusivity during the thin-layer drying of figs. J Food Eng. 2004;65(3):449–58. 10.1016/j.jfoodeng.2004.02.005

[44] Tulek Y. Drying kinetics of oyster mushroom (*Pleurotus ostreatus*) in a convective hot air dryer. J Agric Sci Technol. 2011;13(5):655–64.

[45] Minaei S Motevali A Ahmadi E Azizi MH. Mathematical models of drying pomegranate arils in vacuum and microwave dryers. J Agric Sci Technol. 2012;14(2):311–25.

[46] Doymaz I. Thin-layer drying characteristics of sweet potato slices and mathematical modelling. Heat Mass Transf. 2011;47(3):277–85. 10.1007/s00231-010-0722-3

[47] Elangovan E Natarajan SK. Effects of pretreatments on quality attributes, moisture diffusivity, and activation energy of solar dried ivy gourd. J Food Process Eng. 2021;44(4):e13653. 10.1111/jfpe.13653

[48] Chen XD. Moisture diffusivity in food and biological materials. Dry Technol. 2007;25(7-8):1203–13. 10.1080/07373930701438592

